# Amine‐to‐Azide Conversion on Native RNA via Metal‐Free Diazotransfer Opens New Avenues for RNA Manipulations

**DOI:** 10.1002/anie.202015034

**Published:** 2021-02-18

**Authors:** Olga A. Krasheninina, Julia Thaler, Matthias D. Erlacher, Ronald Micura

**Affiliations:** ^1^ Institute of Organic Chemistry and Center for Molecular Biosciences University of Innsbruck Innrain 80–82 6020 Innsbruck Austria; ^2^ Institute of Genomics and RNomics Biocenter Medical University of Innsbruck Innrain 80–82 6020 Innsbruck Austria

**Keywords:** click chemistry, peptidyl-RNA conjugates, RNA solid-phase synthesis, traceless Staudinger ligation, tRNA

## Abstract

A major challenge in the field of RNA chemistry is the identification of selective and quantitative conversion reactions on RNA that can be used for tagging and any other RNA tool development. Here, we introduce metal‐free diazotransfer on native RNA containing an aliphatic primary amino group using the diazotizing reagent fluorosulfuryl azide (FSO_2_N_3_). The reaction provides the corresponding azide‐modified RNA in nearly quantitatively yields without affecting the nucleobase amino groups. The obtained azido‐RNA can then be further processed utilizing well‐established bioortho‐gonal reactions, such as azide‐alkyne cycloadditions (Click) or Staudinger ligations. We exemplify the robustness of this approach for the synthesis of peptidyl‐tRNA mimics and for the pull‐down of 3‐(3‐amino‐3‐carboxypropyl)uridine (acp^3^U)‐ and lysidine (k^2^C)‐containing tRNAs of an Escherichia coli tRNA pool isolated from cellular extracts. Our approach therefore adds a new dimension to the targeted chemical manipulation of diverse RNA species.

## Graphical abstract

Metal‐free diazotransfer on native RNA using the reagent FSO_2_N_3_ selectively provides azide‐modified RNA in quantitative yields. The stereochemically controlled reaction opens new avenues for the synthesis of RNA‐peptide conjugates and for the isolation of cellular RNA containing post‐transcriptional nucleotide modifications with an aliphatic primary amino group, such as acp^3^U.

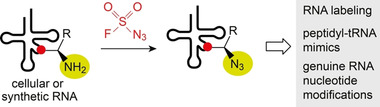

Ribonucleic acid (RNA) is a fragile molecule that does not survive harsh reaction conditions and thus makes selective modification by synthetic organic chemistry challenging.[^1^, ^2^, ^3^, ^4^, ^5^] With only four building blocks (A, C, G, U), RNA is structurally rather uniform and only offers the primary alcohol of the 5′ terminal ribose, and the diol at the 3′‐terminal ribose (2′‐OH, 3′‐OH) as unique structural features for direct selective chemical transformations. Most prominent is the utilization of the diol moiety for labeling of native RNA through periodate cleavage to the corresponding dialdehyde and subsequent attachment of reporter groups (e.g. fluorophores, biotin, etc.) by reductive amination reactions.[^6^, ^7^, ^8^]

The functionalities of RNA are more diverse when one considers naturally occurring RNA with more than 140 modifications known to date, most of them found in tRNA, and also in rRNA, mRNA and non‐coding RNAs.[^9^, ^10^, ^11^, ^12^, ^13^] A broad spectrum of modifications is encountered, ranging from simple methylations to very complex nucleotides containing tricyclic nucleobases (wybutosine), deazanucleobases with sugar moieties attached (queuosine), or amino acid conjugated nucleobases (e.g. lysidine). RNA containing such modifications is endowed with functional groups that are distinct from the repetitive nucleotide pattern of RNA, and therefore, they can serve as handle for specific and selective transformations. Reactive handles can also be generated through metabolic labeling of RNA[^14^, ^15^, ^16^, ^17^, ^18^, ^19^, ^20^, ^21^] or by RNA solid‐phase synthesis.[^22^, ^23^, ^24^, ^25^, ^26^, ^27^, ^28^, ^29^, ^30^]

In the present work, we have searched for a solution to convert an RNA containing a primary amino group into the corresponding azide‐modified RNA, leaving the nucleobase amines unaffected (Figure 1). Finding such a conversion reaction would open new avenues for RNA labeling, for the preparation of RNA‐peptide conjugates, and for the selective isolation and identification of cellular RNAs with nucleotide modifications, such as 3‐(3‐amino‐3‐carboxypropyl)uridine (acp^3^U),^[31]^ lysidine (k^2^C),^[32]^ 5‐aminomethyl‐2‐thiouridine (mn^5^s^2^U),^[9]^ and many others (Supporting Figure S1).^[9]^ The transformation of their primary amines into azides would deliver a well‐behaved reactive handle that allows the application of high‐yielding bioorthogonal conjugation reactions (e.g. azide‐alkyne cycloadditions (Click)[^33^, ^34^, ^35^, ^36^, ^37^, ^38^] or Staudinger ligations[^39^, ^40^, ^41^]).

**Figure 1:**
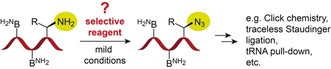
RNA with primary aliphatic amino group modifications of natural or synthetic origin (e.g. tRNAs, aminoacyl‐RNAs, synthetic RNA‐peptide conjugates, etc.) currently suffer from a lack of suitable methods that allow for selective chemical transformation into azide functionalities without affecting the nucleobase NH_2_ groups (B^NH2^).

To reach this goal, our attention was caught by a recently published diazotizing reagent, fluorosulfuryl azide (FSO_2_N_3_), originally designed to provide rapid access to azido libraries of small organic compounds.^[42]^ We considered this reagent promising to tolerate the functionalities present in RNA. Therefore starting from commercially available 1‐(fluorosulfuryl)‐2,3‐dimethyl‐1*H*‐imidazol‐3‐ium trifluoromethanesulfonate,^[42]^ we first optimized the preparation of the diazotizing reagent by implementation of a centrifugation step to achieve a reagent grade suitable for RNA treatment (see Supporting Information). Then, to investigate the diazotransfer reaction between an RNA comprising an aliphatic amino group and FSO_2_N_3_, two 3′‐aminoacylated oligoribonucleotides (5′‐UCCA‐3′‐NH‐Gly‐NH_2_, 5′‐p‐UCCACAGAAUUCGCACCA‐3′‐NH‐Phe‐NH_2_), and a 5‐aminomethyl uridine containing RNA (**1**–**3**) were chosen and individually treated by an excess of FSO_2_N_3_ in a biphasic mixture of methyl *tert*‐butyl ether (MTBE), DMF, and aqueous NaHCO_3_ solution, thoroughly mixed for 20 minutes (Figure 2). After phase separation, the RNA products **1**–**3 az** (in the aqueous phase) were analyzed by ion exchange chromatography. Indeed, all three oligoribonucleotides gave a very clean HPLC trace with a major peak at slightly higher retention time, from which the yields were estimated to be higher than 95 % (Figure 2, Table 1; for further examples (**4**–**7 az**) see Supporting Figure S2 and Supporting Table S1). The integrity of the products was confirmed by mass spectrometry (Figure 2). We mention that a control RNA, 5′‐AACGAGGCCACAGG, possessing only the exocyclic amino groups at the nucleobases (and no aliphatic primary amine) remained unaffected under the same reaction conditions (Supporting Figure S3). Further, we note that the diazotransfer reaction retains the stereochemical configuration of the amino substituted carbon of the substrate.^[43]^ A primary attack of the amino nucleophile at the terminal nitrogen of the N_3_ substituent resulting in transfer of the two terminal nitrogen atoms of the azide to the product was verified in an early mechanistic study on the closely related reagent, imidazole‐1‐sulfonyl azide.[^43^, ^44^] Therefore, the diazotransfer reaction becomes highly valuable for application in RNA‐peptide conjugation chemistry as described further below.

**Figure 2:**
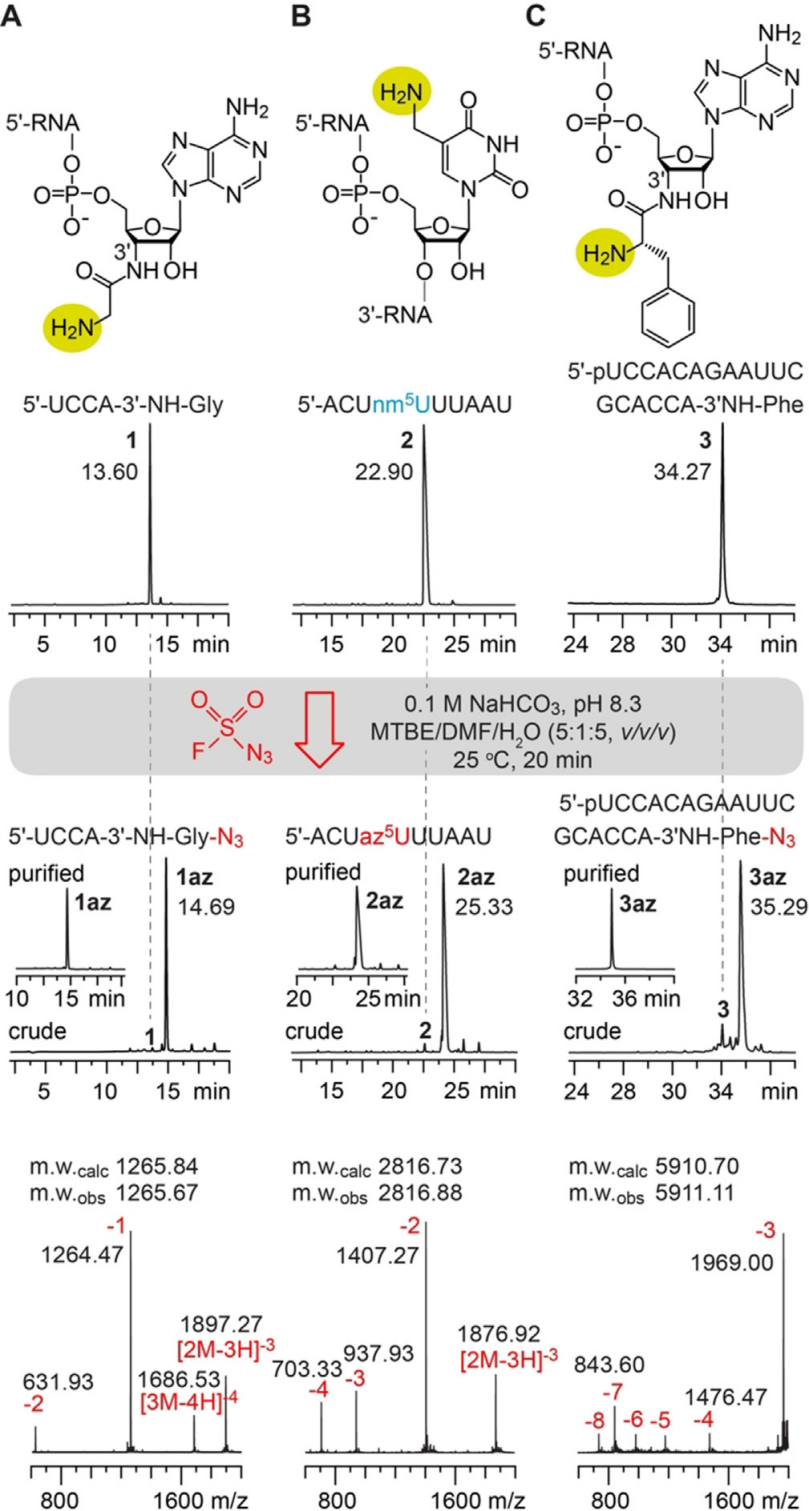
Diazotransfer reactions on RNAs containing an aliphatic primary amino group using the FSO_2_N_3_ reagent. A) The amino terminus of the short glycyl‐tRNA mimic reacts quantitatively to form the corresponding azide using the reagent under the conditions indicated. B) Same as (A) but for a 5′‐aminomethyluridine (nm^5^U)‐containing RNA. C) Same as (A) but for an 18 nt l‐phenylalanyl‐tRNA mimic with a sterically hindered α‐amino group, under retention of configuration; anion‐exchange HPLC traces of crude and purified conjugates (middle), and corresponding LC‐ESI mass spectra (bottom).

**Table 1:** Overview of selected amine‐to‐azide converted oligoribonucleotides and conjugates.^[a]^

No.	Oligoribonucleotides^[b]^	Reaction yields^[c]^ [%]	m.w._obs_^[d]^ [amu]	m.w._calc_ [amu]
**1 az**	UCCA‐3′‐NH‐Gly‐N_3_	98	1265.67	1265.84
**2 az**	ACUazm^5^UUUAAU	98	2816.88	2816.73
**3 az**	pUCCACAGAAUUCGCACCA‐3′‐NH‐Phe‐N_3_	95	5911.11	5910.70
**4 az**	CAGUUGACUazm^5^UUUAAUCAAUUG	96	6675.40	6675.03
**5 az**	UUCCCUUCGCCCGCUCCA‐3′‐NH‐Gly‐N_3_	91	5622.90	5622.29
**6 az**	UCCCGUCAUCACCCACCA‐3′‐NH‐Val‐N_3_	92	5694.04	5694.57
**7 az**	GUCCACUCAGGCCUACCA‐3′‐NH‐Ile‐N_3_	96	5789.20	5788.65
**2 az⋅biotin**	ACUbiotin‐m^5^UUUAAU	98	3274.40	3274.31
**3 az⋅fMet**	pUCCACAGAAUUCGCACCA‐3′NH‐Phe‐fMet	60	6044.80	6043.90
**5 az⋅fMet**	UUCCCUUCGCCCGCUCCA‐3′‐NH‐Gly‐fMet	71	5756.13	5756.63
**6 az⋅fMet**	UCCCGUCAUCACCCACCA‐3′‐NH‐Val‐fMet	79	5828.03	5827.78
**7 az⋅fMet**	GUCCACUCAGGCCUACCA‐3′‐NH‐Ile‐fMet	86	5922.25	5921.86

[a] For the complete list see the Supporting Information. [b] Oligonucleotide sequence in 5′ to 3′ direction and peptide sequence from C to N terminus; [c] determined from areas in HPLC profiles; [d] molecular weights m.w. obtained by LC‐ESI ion trap mass spectrometry.

With the highly efficient RNA amine‐to‐azide conversion in hand, we set out to demonstrate the applicability of the so generated azido‐RNAs for bioconjugation reactions and first utilized the most popular one, the copper(I)‐catalyzed alkyne‐azide cycloaddition (CuAAC).[^33^, ^34^, ^35^, ^36^, ^37^, ^38^] Using a typical CuAAC reaction setup with CuSO_4_, ascorbic acid and tris(3‐hydroxypropyltriazolylmethyl)amine (THPTA) as Cu^I^ stabilizing ligand, we reacted the crude product of the 5‐azidomethyluridine modified RNA **2 az** that was obtained after amine‐to‐azide conversion with a biotin‐alkyne derivative (Figure 3 A). Efficient biotin attachment was reflected in a major peak of the corresponding anion exchange HPLC chromatogram of the reaction mixture, and after HPLC purification the integrity of the triazole‐linked RNA‐biotin conjugate **2 az⋅biotin** was confirmed by mass spectrometry (Figure 3 A, Table 1; for further examples, **1 az⋅biotin** and **4 az⋅biotin**, see Supporting Figure S4 and Supporting Table S1).

**Figure 3:**
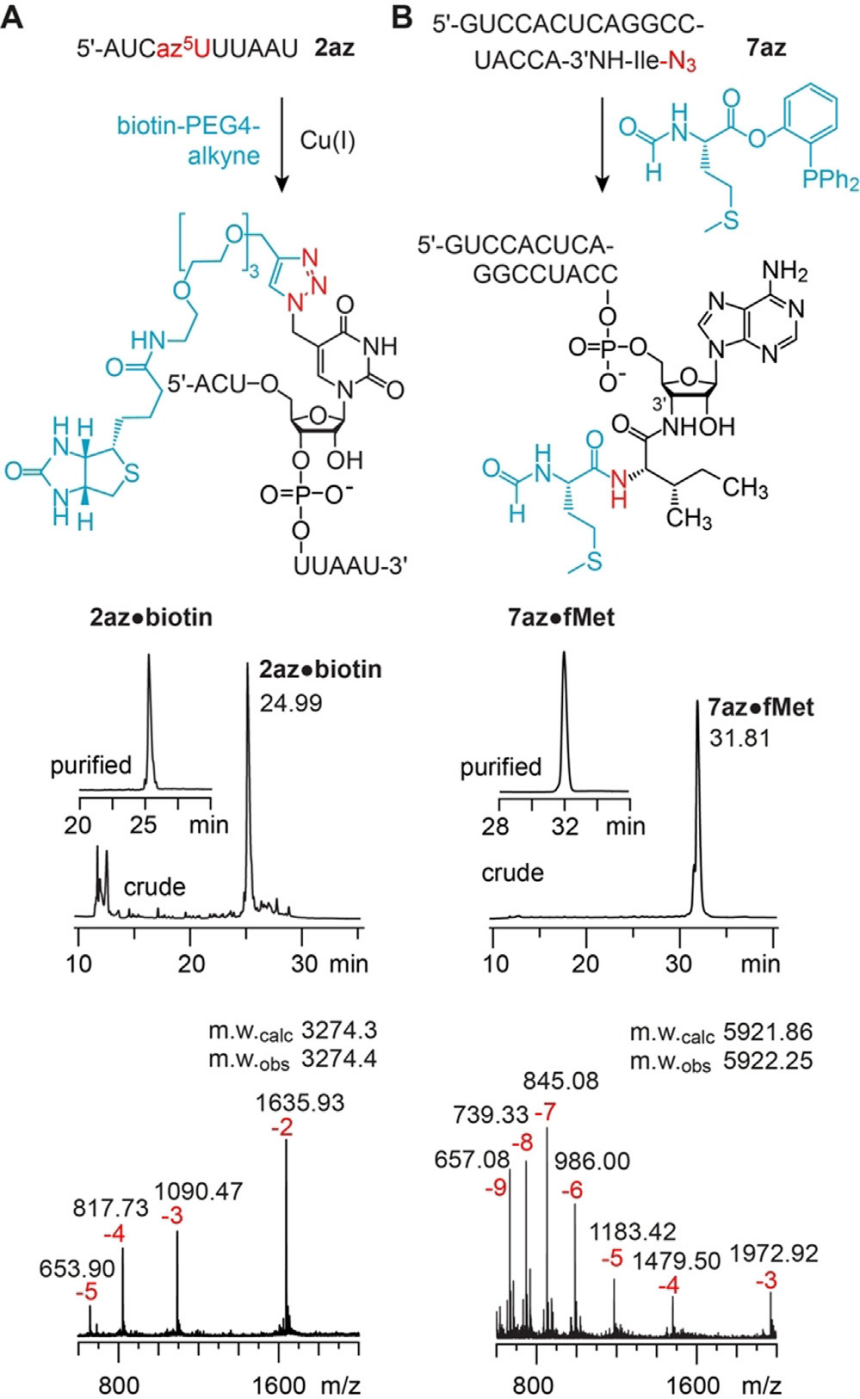
Applications of azido‐RNAs obtained by diazotransfer reaction with FSO_2_N_3_. A) Copper(I)‐catalyzed alkyne‐azide cycloaddition exemplified for an az^5^U‐modified RNA (**2 az**); B) Traceless Staudinger ligation exemplified for a mimic of l‐isoleucine charged *E. coli* tRNA^Ile^ (**7 az**); analytical anion‐exchange (AE) HPLC traces of crude and purified conjugates (middle), and LC‐ESI mass spectra (bottom). Purification was performed on a semipreparative AE column using gradient elution with a flat slope (for details see Supporting Information).

A true benefit of the here introduced amine‐to‐azide conversion on native RNAs arises for the synthesis of peptidyl‐tRNA mimics.[^45^, ^46^, ^47^, ^48^, ^49^] These mimics are needed for cryo‐EM and X‐ray crystallographic studies of ribosomal translation,[^50^, ^51^] and for a variety of biochemical approaches to explore translation phenomena, such as ribosome stalling induced by macrolide antibiotics,^[52]^ or slow peptide bond formation when proline^[53]^ or d‐amino acids are brought to the ribosome active site.^[54]^ We have previously shown that hydrolysis‐resistant 3′‐peptidyl‐tRNA mimics are accessible by native chemical ligation starting from l‐cysteinyl‐3′‐amino‐RNA.^[55]^ The approach, however, is limited to cysteine at the ligation site, and only alanine and valine are accessible in addition, when desulfurization procedures are applied.^[56]^ This limitation falls with the amine‐to‐azide conversion chemistry introduced in this work. In combination with our previously established routes to any desired aminoacyl‐3′‐amino‐RNA,[^57^, ^58^] we can tap the full potential of methods alternative to native chemical ligation. In particular, these are traceless‐Staudinger ligations,[^59^, ^60^, ^61^, ^62^] first described in the year 2000 by the groups of Bertozzi^[59]^ and Raines,^[62]^ that employ an azide and a phosphine, reacting to an iminophosphorane (aza ylide) that intramolecularly attacks an ester or thioester. After hydrolysis of the cyclic intermediate, a native amide bond is obtained (Supporting Figure S5).

For the above reasons, we set out to demonstrate the feasibility of traceless Staudinger ligations on the example of azido‐l‐isoleucyl‐RNA **7 az** that mimics the 3′‐terminal sequence of tRNA^Ile^ from *E. coli* (Figure 3 B) and that was generated by amine‐to‐azide conversion using FSO_2_N_3_ (Figure 2 C). To the best of our knowledge, traceless Staudinger ligations on RNA substrates have not yet been described in the literature, and we believe that this is due to poor solubility of native RNAs in (organic) solvents that are typically used for these ligations. We circumvented this problem by converting the azido‐RNA to a form that is soluble in organic solvents by precipitation with a quaternary ammonium surfactant, cetyltrimethylammonium bromide (CTAB). The CTAB‐treated azido‐l‐isoleucyl‐RNA **7 az** was then reacted with *N*‐formyl‐l‐methionine 2‐(diphenylphosphino)phenyl ester (for preparation see Supporting Information) to yield **7 az⋅fMet** (Figure 3 B and Supporting Figure S6). The ligation conditions were optimized; the highest yields (up to 86 %) were obtained with high concentrations of substrate and phosphine (≈50 μm of RNA and 0.1 m of phosphine) and the use of wet DMF at 60 °C after 4 h. We confirmed the robustness of the ligation by additional examples using azido‐l‐phenylalanyl‐, azido‐glycyl‐, and azido‐l‐valyl‐RNAs (**3 az**, **5 az**, and **6 az**, respectively) to give **3 az⋅fMet**, **5 az⋅fMet**, and **6 az⋅fMet**, respectively (Table 1, Supporting Figure S4).

To further demonstrate the value of the amine‐to‐azide conversion we turned to RNA from natural sources. Our goal was to enrich and analyze RNAs that contain native modifications which possess an aliphatic primary amino group, from a cellular RNA pool. We decided to focus on *E. coli* tRNAs for which the modifications 3‐(3‐amino‐3‐carboxypropyl)uridine (acp^3^U) and lysidine (k^2^C) are known (Supporting Figures S1 and S7).[^9^, ^13^] The acp^3^U is a very common modified nucleotide found in the dihydrouridine (D) and variable (V) loops of tRNAs, however, little is known about the physiological functions.[^31^, ^63^, ^64^] In *E. coli*, acp^3^U resides in the V‐loop at position 47 of tRNA^Lys^, tRNA^Met^, tRNA^Arg^, tRNA^Phe^, tRNA^Ile1^, tRNA^Ile2^, tRNA^Ile3^, tRNA^Val1^, and tRNA^Val2^, while k^2^C is located in the anticodon loop at position 34 of tRNA^Ile3^ only (Supporting Figures S7 and S8).[^9^, ^32^]

Our strategy to utilize a combination of diazotransfer and Click chemistries for a pull‐down assay of tRNAs containing acp^3^U47 and k^2^C34 from the total *E.coli* tRNA pool is schematically shown in Figure 4 A–C. The first step is treatment of the tRNAs with FSO_2_N_3_ under the above optimized conditions to convert all aliphatic primary amino groups into azides. Then, CuAAC with desthiobiotin‐PEG4‐alkyne is applied, based on the conditions optimized for the short az^5^U oligoribonucleotide shown above. Subsequently, the resulting tRNA reaction mixture is subjected to streptavidin covered magnetic beads (SMBs) to immobilize the tRNA fraction that has been labeled with desthiobiotin. After several washing steps to remove unlabeled tRNAs, the labeled tRNA fraction is eluted with a biotin solution under non‐denaturing conditions at room temperature. In order to identify the individual tRNAs, we carried out northern blotting. Indeed, all tRNA species annotated with the acp^3^U47 modification and tRNA^Ile3^ annotated also with the k^2^C34 modification were unequivocally confirmed in the enriched pool (Figure 4 D). As negative control, tRNA^Gly^ having no modifications with aliphatic primary amino groups was not detectable (Figure 4 D, lane 2). As a marker of RNA length, we used in vitro transcribed tRNA^Lys^, depicted as first lane in Figure 4 D. Taken together, this protocol defines a robust method for the pull‐down of cellular RNAs that contain a modification with an aliphatic primary amino group, and therefore this approach forms a solid basis for investigations that aim at the elucidation of the physiological functions of these particular nucleotide modifications.

**Figure 4:**
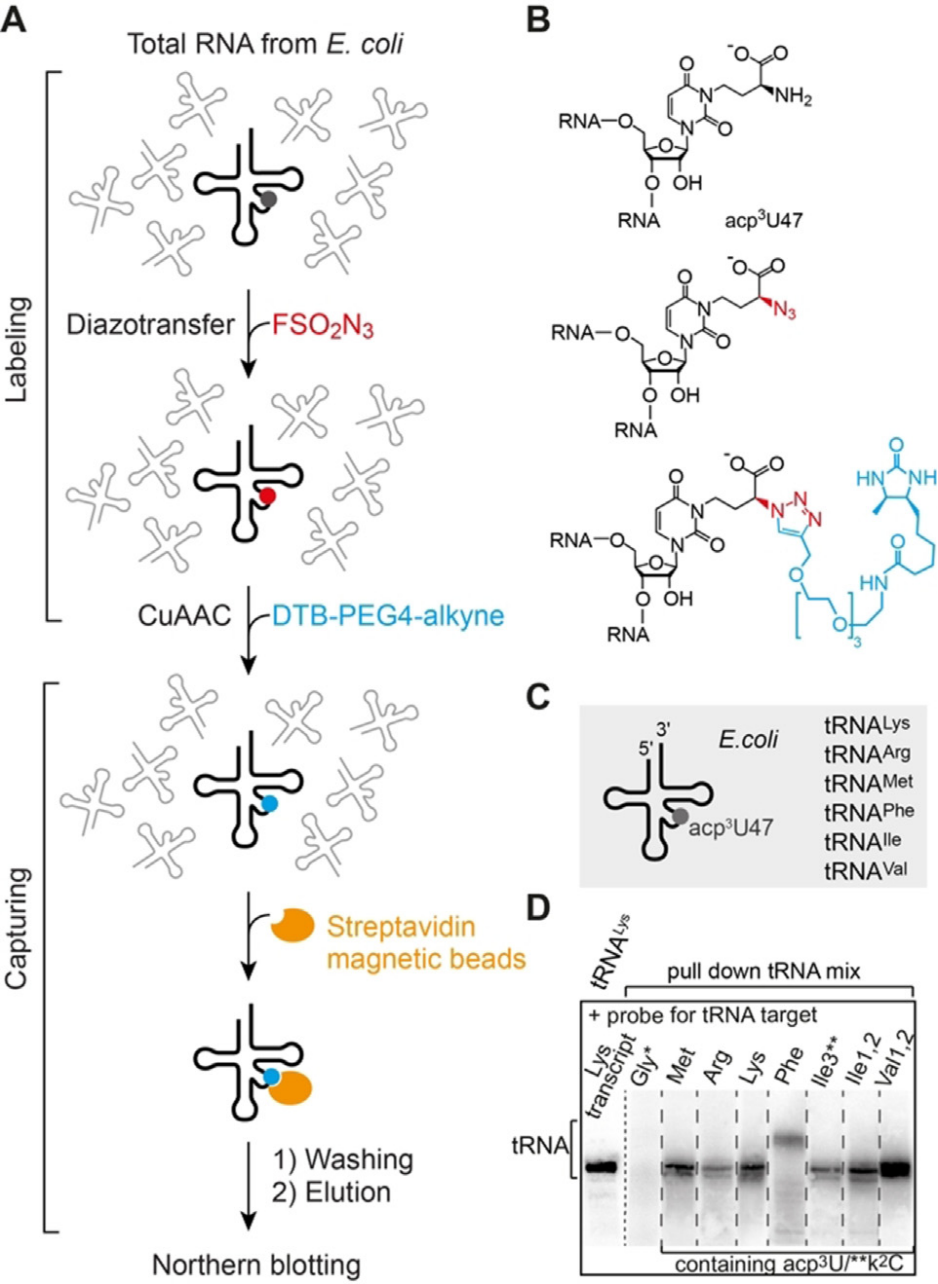
Pull‐down of tRNAs containing native modifications with aliphatic primary amino groups. A) Experimental concept for selective amine‐to‐azide transfer by FSO_2_N_3_ and labeling by CuAAC with desthiobiotin‐PEG4‐alkyne, followed by capturing, elution, and analysis of the enriched tRNA pool. B) Chemical structure of 3‐(3‐amino‐3‐carboxypropyl)uridine (acp^3^U) and the corresponding products after the diazotransfer and labeling. C) Schematic representation of the *E. coli* tRNAs containing acp^3^U at position 47 (for full sequence information see Supporting Figure S7). D) Northern blot analysis was used for the identification of individual tRNAs in the enriched *E. coli* tRNA mixture. *Probes selective for tRNA^Gly^ without a primary amino group modification served as negative control, as expected tRNA^Gly^ was not detected. **Besides acp^3^U47, *E. coli* tRNA^Ile3^ also contains k^2^C34 (lysidine). In vitro transcribed tRNA^Lys^ (without modifications) was used as marker of RNA length.

In summary, our new approach for selective amine‐to‐azide conversions on RNA targets containing primary amino functionalities paves the way to RNA bioconjugation approaches that make use of the most efficient biorthogonal reactions known to date for azido compounds. In this context, we point out that the direct labeling of a sterically hindered primary amino group by using active ester[^65^, ^66^, ^67^] or isocyanate^[68]^ reagents is usually troublesome and inferior to the here introduced straightforward two‐step procedure because of low yields and tedious optimization of pH‐ and salt‐dependent reaction conditions. We are confident that the new diazotransfer reaction with the FSO_2_N_3_ reagent will significantly expand the repertoire of bioorthogonal RNA bioconjugation chemistry, enabling exciting new biochemical applications ranging from the chemically controlled spatial and temporal activation of RNAs to the direct manipulation of metabolically labeled RNAs.

## Conflict of interest

The authors declare no conflict of interest.

## Supporting information

As a service to our authors and readers, this journal provides supporting information supplied by the authors. Such materials are peer reviewed and may be re‐organized for online delivery, but are not copy‐edited or typeset. Technical support issues arising from supporting information (other than missing files) should be addressed to the authors.

Supplementary
